# Upper airway flow characteristics of childhood obstructive sleep apnea-hypopnea syndrome

**DOI:** 10.1038/s41598-022-10367-w

**Published:** 2022-05-05

**Authors:** Huikun Cai, Chang Xu, Haoyang Xue, Yufeng Guo, Lijun Su, Xingqiang Gao

**Affiliations:** 1 Department of Mechanical and Electrical Engineering, Xiamen University, No. 4221-134, Xiangan South Road, Xiangan South District, Xiamen City, 361102 Fujian Province China; 2 Children’s Hospital of Xiamen, Xiamen City, 361006 Fujian Province China

**Keywords:** Biomedical engineering, Mechanical engineering

## Abstract

Revealing the structural morphology and inner flow field of the upper airway is important for understanding obstructive sleep apnea-hypopnea syndrome (OSAHS) incidence phenomena and pathological diagnosis in children. However, prior work on this topic has been focused on adults and the findings cannot be directly extrapolated to children because of different inducing factors. Therefore, this paper employs a simulation method to investigate upper airway flow characteristics of childhood OSAHS. It is found that the Reynold number changes highly throughout the whole upper airway, and the laminar assumption is no longer suitable for low Reynold number flow, which is much unlike classic fluid mechanics. Turbulent models of Standard k-ω and Spalart-Allmaras were developed prior to suggestion. The simulation is validated by experiments with an error of approximately 20%. Additionally, carried out in this analysis is the influence of adenoidal hypertrophy with different narrow levels. The cross-sectional area, flow velocity, pressure drop and volume rate will change greatly when the narrow level is above 64% of the upper airway, which can be a quantitative explanation for medical intervention if adenoid hypertrophy blocks 2/3 of the upper airway in the common clinical judgment of otorhinolaryngology. It is expected that this paper can be a meaningful instruction on OSAHS surgery plan making as well as recovery evaluation postoperatively.

## Introduction

Obstructive sleep apnea–hypopnea syndrome (OSAHS) can greatly affect children’s physical and psychological health in terms of craniofacial anomalies, cardiovascular disease, retinopathy, lymphoid hyperplasia and maldevelopment ^1–3^, and its prevalence has increased to 8% since 2005 ^4^. Traditional diagnosis is always based on medical imaging technology, such as computed tomography (CT) and magnetic resonance imaging (MRI), as these medical images can clearly show structural abnormities in the upper airway. However, further pathology mechanism analysis, such as flow and pressure disorders caused by structural abnormities, cannot be gained from medical images. The analysis cannot also be gained from clinical nasal resistance test in vitro, as the test can only measure nasal pressure but not detailed inner flow status of the upper airway.

Benefiting from the development of computational fluid dynamics (CFD), the flow characteristics of the upper airway can be fully captured in terms of pressure, velocity and mass flow rate so that CFD method is increasingly used in the study of OSAHS. The CFD method is an interdisciplinary subject among mathematics, fluid mechanics and computer science that has emerged since the 1950s with the development of computers. It adopts finite difference method and finite element method to get numerical solutions of flow governing Navi-Stokes equations under the help of computer. The method has generally derived a variety of optimized physical models, such as steady and unsteady flow, laminar and turbulent flow, incompressible and compressible flow, and so on. For flow characteristics of each physical problem, explicit or implicit difference scheme can be chosen to achieve the best balance among calculation speed, stability and accuracy, and the solutions have been proved quite reliable in many engineering practice ^5,6^. An initial trial was carried out in adults and proved that CFD was a potentially useful modality for the clinical assessment of OSAHS medical properties. In contrast to adults, the main factors inducing OSAHS in children are adenoid and tonsillar hypertrophy but not soft palate overlong or blocked-up ^7–9^. Consequently, children’s  upper airway structure, clinical manifestation and therapeutic regimen are also different from those of adults, and the research achievement of adult OSAHS cannot be directly extrapolated to childern. Therefore, revealing the structural morphology and flow characteristics of the child upper airway has become a new interesting and hot topic, as it is important and meaningful for the understanding of childhood OSAHS incidence phenomena and pathological diagnosis.

For childhood OSAHS, Raanan et al. ^10,11^ studied the differences in upper airway structure with and without OSAHS and found that in children with moderate OSHAS, the upper airway was restricted both by adenoids and tonsils as well as the soft palate. However, they did not show a flowing process or flowing field in the upper airway. Chun et al. ^12^ used CFD to analyze  the effect of airway geometry on internal pressure in the upper airway of three children with OSAHS and three controls and suggested that pharyngeal airway shape in children with OSHAS significantly affected internal pressure distribution compared to nasal resistance, but the model was not a whole upper airway model, as they did many simplifications on nasal structure that could influence pressure and flow fields. Luo et al. ^13^ built models of the upper airway from the nares to trachea before and after adenotonsillectomy (AT), and CFD results showed that the apnea hypopnea index decrease after AT was strongly correlated with a reduction of the maximum pressure drop in the region where the tonsils and adenoid constricted the pharynx. Zhao et al. ^14^ analyzed the effect of orthodontic treatment on OSAHS in CFD simulations and found that orthodontic treatment could expand airway volume from the palatopharynx to the glossopharynx, lower negative pressure and flow resistance of narrow areas in the upper airway and had a positive influence on OSAHS. Slaats et al. ^15^ reconstructed a 3D model from functional respiratory imaging (FRI) combined with CFD, and used a linear solution to solve the flowing process. They found that children with more severe OSA had a smaller volume of the overlap region between the adenoids and tonsils. However, these simulations need more experimental validations.

As mentioned above, research on the upper airway structure, clinical manifestation and therapeutic regimen of child OSAHS has gained many results, but there are still many problems that need solutions by numerical and experimental investigations. One of the problems is that there is no convincing numerical method for inner flow field research. Due to the simplified structure of the upper airway, different CFD models were applied in the simulations and gained different results, some of which disagreed greatly with the other in values or even inversed in tendency. Another problem is that many simulations lack experimental validations to prove model rationality, as traditional validations are limited greatly in terms of accuracy, reliability, simplicity, convenience and operability. Therefore, this paper presents a complete model of the child’s upper airway starting from the anterior naris and ending at the beginning of the trachea. CFD simulations with different solving models are carried out and validated by lab experiments using 3D printing technology. It is expected to gain the thorough flow characteristic and pressure field of the upper airway and can be helpful for understanding the pathological mechanism of childhood OSAHS.

## Methods and theories

The study was approved by the Ethics Committee of Children's Hospital of Xiamen, China, and all methods were performed in accordance with the relevant guidelines and regulations. Informed consent was obtained from parents of volunteer subjects. As shown in Table 1, control subject with normal growth and no abnormalities diseases was matched to subject with OSAHS by age, sex, ethnicity, weight, and height. Before scanning, the testees should be awake and sit quietly for ten minutes, and then lie flat to ensure a smooth and peaceful breath.

**Table 1:** Detailed clinical data of children with and without OSAHS.

Subjects	Sex	Age	Height/cm	Weight/kg	AHI of PSG/pcs/h	LSaO2 of PSG	OSAHS grading
Child with OSAHS	Boy	Six years and one month	115	19	27	70%	Serious
Child without OSAHS	Boy	Six years and five months	117	21	2	90%	Normal

### Model reconstruction and CFD simulation setting

**Figure 1:**
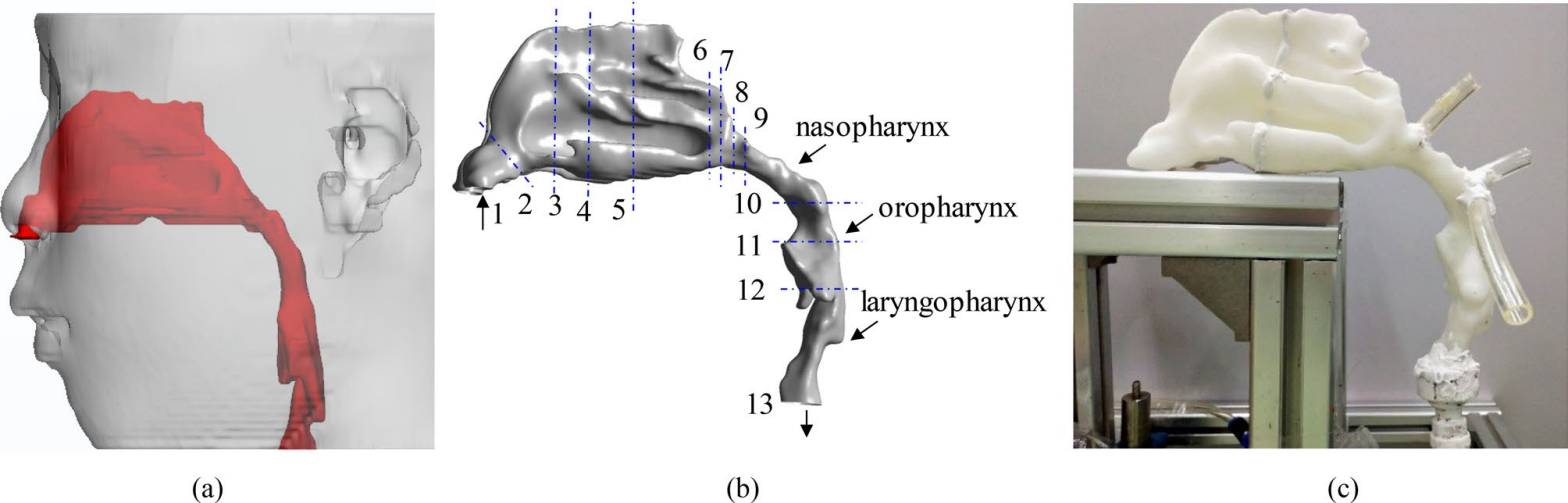
(**a**) is a schematic diagram of the upper airway in the body; (**b**) is the 3D simulation model reconstructed by the CT scanning image; and (**c**) is the 3D printing model for the lab experiment.

The simulation and experiment were firstly carried out on control subject without OSAHS to confirm the analysis method and study basic inner flow characteristics. A upper airway usually included the nasal, nasopharynx, oropharynx, laryngopharynx and glottis. Therefore, CT images from the anterior naris to tracheal beginning with a scanning layer spacing of 1.25 mm were imported into commercial software (Amira 3.0, TGS, Inc.) for a 3D model reconstruction. Airway cross-sections perpendicular to the airway direction were defined at different locations, and thirteen cross-sections were shown in Table 2 and Fig. 1. To improve grid quality, Materialise 3-matic software was used to optimize the qualities of triangle slices in the *.stl file. The optimization process was as follows: (1) homogenized triangle slices; (2) built well-flat airway inlet and outlet; (3) refined triangle slices in calculating core area; (4) checked model quality to ensure that there was no overlap, intersection, hole and bad edges existing in triangle slices.

**Table 2:** Thirteen cross-sections of upper airway.

Cross-section	Location	Cross-section	Location	Cross-section	Location
1	Anterior naris	6	End Inferior turbinate	11	Tonsil midsection
2	Nasal limen	7	Posterior naris	12	Hypopharyngeal beginning
3	Anterior inferior turbinate	8	Nasopharyngeal beginning	13	Tracheal beginning
4	Anterior middle turbinate	9	Adenoid	–	–
5	Middle inferior turbinate	10	Oropharyngeal beginning	–	–

After optimization, the 3D model was imported into commercial CFD meshing software (ICEM, ANSYS, Lebanon, NH, USA) to create unstructured tri/tetrahedral meshes. To capture precise flow characteristics near the wall surface, the mesh in this area was refined with a grid of 10 prismatic layers. The grid height of the first layer should be adjusted to ensure that the *y*^+^ value of the wall surface was small or close to 1 ^16^. The model was solved by commercial finite volume CFD software (Fluent, ANSYS, Lebanon, NH, USA). The inlet and outlet both adopted pressure boundary conditions, and inlet pressure was assumed to be zero on inspiration. For continuity and momentum coupling, the solver used the segregated/implicit type and adopted the SIMPLE algorithm to solve the coupled analyses of speed and pressure. The type of discrete equation adopted a second-order upwind scheme. Convergence in all cases was declared only when both strict criteria were satisfied: (a) reduction in all residuals of at least five orders of magnitude; (b) no observable change in surface temperature prediction for an additional tens of iterations.

### Lab experiment by 3D printing technology

According to 3D printing analysis ^17–19^, photosensitive resin was used as the material of the upper airway wall, and stereolithography apparatus technology was adopted in 3D printing. The upper airway model of 3D printing with a wall thickness of 0.2 mm was of 200% scale to the simulation model. The scaled model was imported into the Materialise Magics software and its printing position was adjusted for slicing. Then the scanning path was also designed here. The laser beam irradiated to the surface of photosensitive resin in accordance with the scanning path, so that a layer of resin in a specific area of the surface was cured. When a layer was processed, a section was generated. Descended the lifting platform to a certain distance, the resin was covered on the first curing layer and the second layer begun to scan until the second curing layer was bonded on the previous curing layer. After layer upon layer, finally the required 3D-printing model was formed and used in lab experiment.

**Figure 2:**
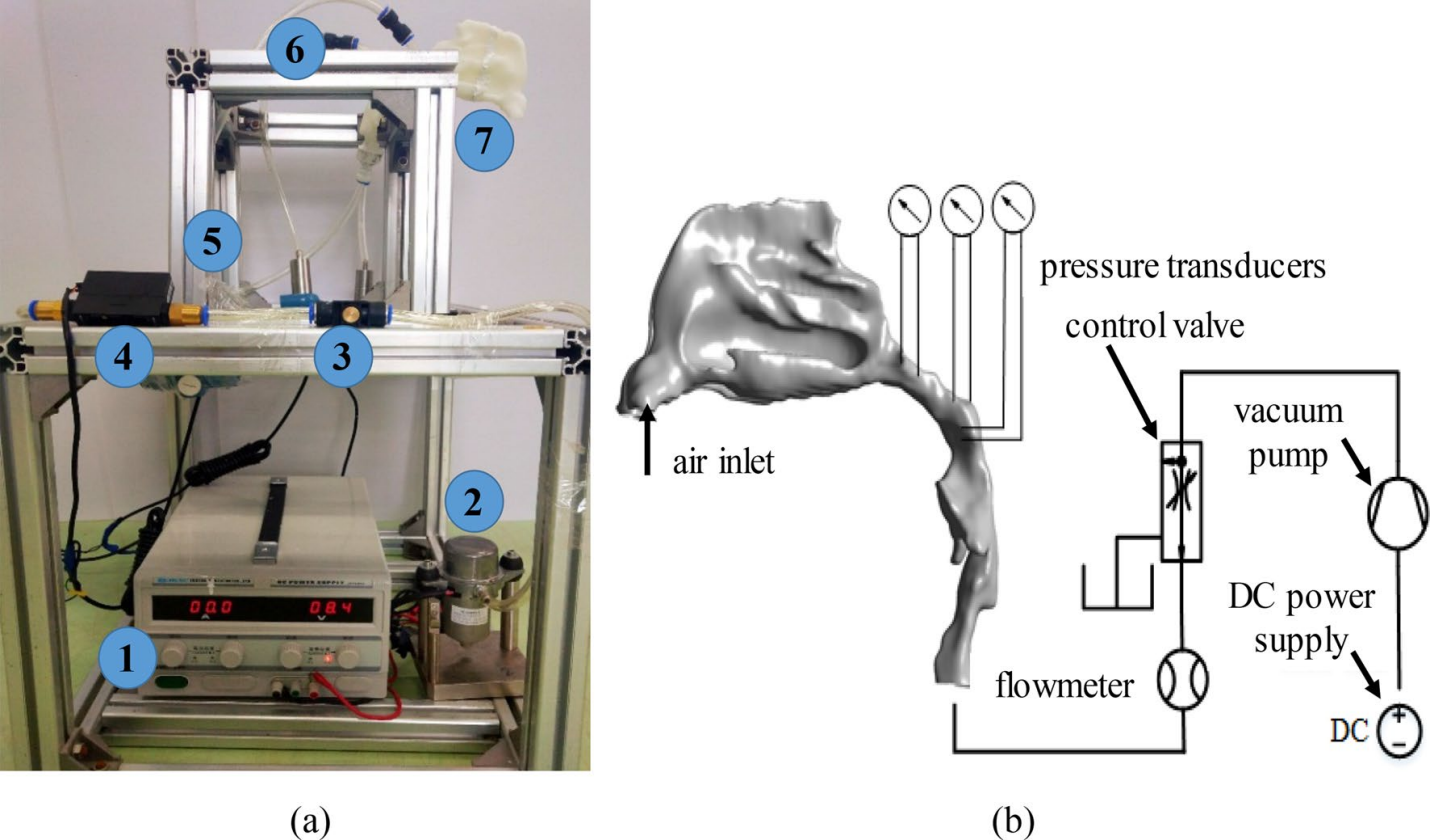
Illustration of experimental validation. (**a**) is the picture of the 3D printing lab experiment, where 1 is the DC power supply, 2 is the vacuum pump, 3 is the control valve, 4 is the flowmeter, 5 is the pressure transducer, 6 is the impulse tube and 7 is the 3D printing model of the upper airway. (**b**) Schematic diagram of lab validation by 3D printing.

As shown in Fig. 2, a vacuum pump was placed at the outlet of the upper airway (tracheal beginning) as an air exhauster to simulate the inspiration process. The inspiration volume was controlled by the pump working voltage and a flow valve was placed between the vacuum pump and airway outlet. Two pressure transducers were placed at the nasopharyngeal part (Transducer 1 located in Cross-section 8 and Transducer 2 located between Cross-sections 9 and 10), and Transducer 3 was placed at the oropharyngeal part (Cross-section 10) to measure the pressures of significant points in the upper airway, as the differences of different flow models were almost concentrated here, and the phenomenon of adverse pressure was also observed in this part according to numerical simulation. The pressure transducer was with a measuring range of − 100–100 Pa and a measuring precision of 1% FS. A flowmeter with a measuring range of 0–50 L/min and measuring precision of 1.5 ± 0.2% FS was used to measure the flow volume rate. Before the experiment, soup suds were smeared on the 3D printing model and junctions to check system air impermeability, and the left nostril was plugged up, which was similar to the nasal resistance test in vitro.

Dynamic similarity between the 3D printing model and simulation model was maintained. As the 3D printing model was 200% scale of the simulation model, its characteristic diameter *D*_*3D*_ was twice to that of the simulation model *D*_*si*_ (*D*_*3D*_: *D*_*si*_ = 2:1). Consequently, to keep the same Reynolds number *Re*, the velocity of 3D printing model *v*_*3D*_ would be half of the simulation velocity *v*_*si*_ (*v*_*3D*_: *v*_*si*_ = 1:2), referring to formula *Re* = *ρvD/η*,where *ρ* is fluid density, *v* is fluid velocity, *D* is fluid characteristic diameter and *η* is fluid viscidity. According to the formula *Q* = *vπd*^*2*^*/4*, the proportion of flow volume in the 3D printing model to that of the simulation model was 2:1 (*Q*_*3D*_: *Q*_*si*_ = 2:1). According to classic fluid mechanics ^20^, the pressure relationship between the 3D printing model and simulation model was *p*_*3D*_ = *p*_*si*_*(ρv*^*2*^*)*_*3D*_*/(ρv*^*2*^*)*_*si*_, and the pressure of the 3D printing model was quarter to that of the simulation model (*p*_*3D*_: *p*_*si*_ = 1:4).

## Calculations and results

### Reynold number calculation

In fluid mechanics, Reynolds number is a dimensionless number used to characterize fluid flow type. When Reynolds number is small, viscous force has greater influence on flow field than inertia force, and velocity disturbance in flow field will attenuate due to viscous force, so that fluid flow is stable and laminar. On the contrary, when Reynolds number is large, the influence of inertia on flow field is greater than that of viscous force, fluid flow is more unstable, and slight change of velocity is easy to develop and enhance, forming a chaotic and irregular turbulent flow field. The laminar and turbulent flow will be solved by different governing equations and numerical models will also different in the simulation. Therefore, in CFD analysis, *Re* should be firstly calculated to estimate the fluid flow is laminar or turbulent. As shown in Fig. 1, the shapes of thirteen cross-sections of the upper airway are varied and irregular; as a result, their *Re* values will vary during the whole flowing process. Based on volume flow formula ^12^:

1 $$\mathrm{Q}=\left(\left(0.019*\mathrm{age\,in\,years}\right)+0.014\right)*1000 \; (\mathrm{mL}/\mathrm{s})$$

**Figure 3:**
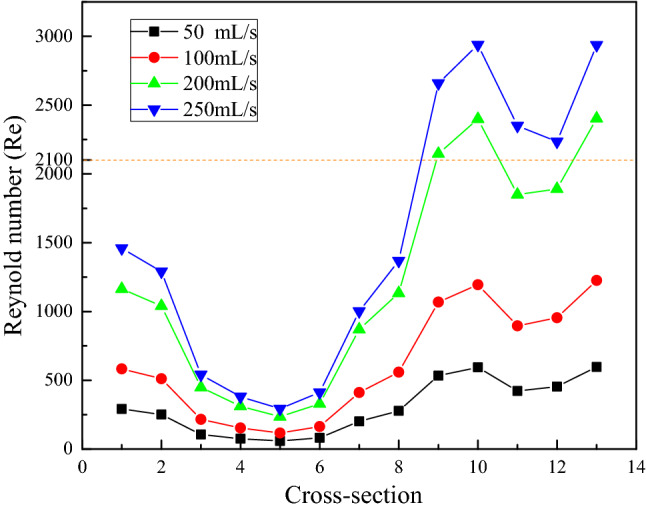
Reynold numbers of thirteen cross-sections vs. different mass flow volumes.

The flow volume of children aged between 2 and 10 years old ranges from approximately 50 to 250 mL/s, and Reynold numbers of thirteen cross-sections with flow volume can also be calculated and are shown in Fig. 3. For flow volumes of 50 and 100 mL/s, the *Re* numbers in thirteen cross-sections are all smaller than 2100. However, for mass volumes of 200 and 250 mL/S, the *Re* numbers can be larger than 2100 beginning in Cross-section 9 due to the area decrease in these cross-sections and increase to the maximum value of 2938 in Cross-section 10 (oropharyngeal beginning) of 250 mL/s. The average *Re* values for the whole upper airway of 200 and 250 mL/s are approximately 1248 and 1528, respectively. In classic fluid mechanics ^20^, usually the flow is assumed to be linear if its Reynold number is below 2100, whereas it is turbulent flow if its Reynold number is above 4000. However, it cannot converge at 200 mL/s when the linear model is adopted in the CFD simulation, regardless of its average *Re* number is smaller than 2100. Therefore, numerical analysis of different flow models with different flow volumes is implemented next.

### Flow distributions of different simulation models

As mentioned above, according to *Re* fluid flow can be divided into laminar or turbulent flow. Laminar flow is stable and linear, and its streamline is usually parallel or almost parallel to flow channel. Whereas turbulent flow is chaotic and irregular, it will develop radial pulsation and vortex. Due to turbulence complexity, an accurate analytic solution cannot be gained so that different numerical solutions are derived by different researchers according to experiments in engineering practice but none of them can suit every condition. The Spalart–Allmaras model is a relatively simple one-equation model that solves a modeled transport equation for the kinematic eddy (turbulent) viscosity. Two-equation models are historically the most widely used turbulence models in CFD. They solve two transport equations and model the Reynolds Stresses using the Eddy Viscosity approach. The standard k-ω model in ANSYS Fluent falls within this class of models. The ω-equation offers several advantages relative to the ε-equation. The most prominent one is that the equation can be integrated without additional terms through the viscous sublayer. This makes the formulation of a robust y^+^-insensitive treatment relatively straightforward.

**Figure 4:**
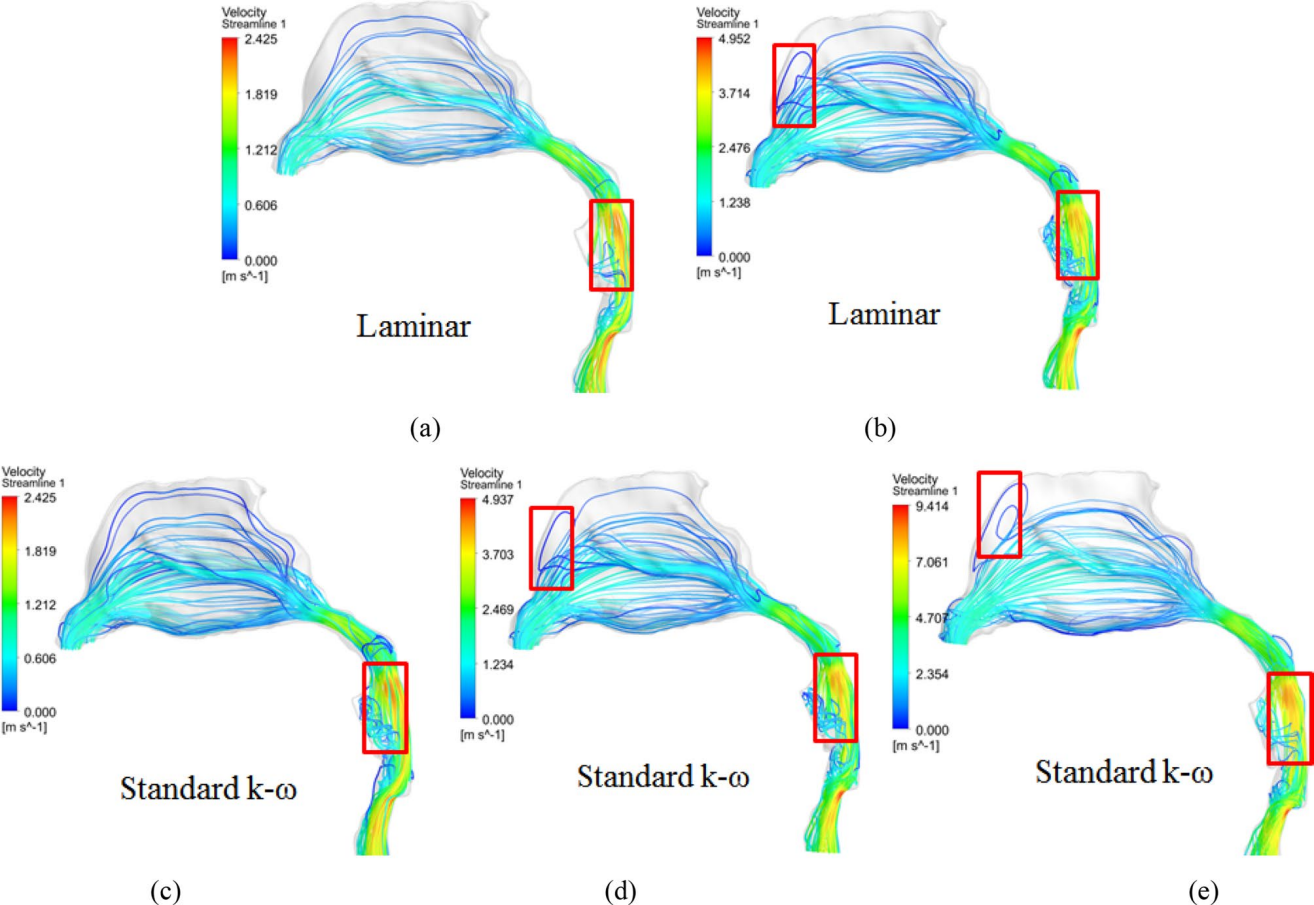
Flow distributions by different simulation models in the upper airway: (**a**) and (**b**) are laminar flow models with flow volumes of 50 and 100 mL/s, respectively; (**c**), (**d**) and (**e**) are turbulent models adopting the standard k-ω style with flow volumes of 50, 100 and 200 mL/s, respectively.

As shown in Fig. 4, it can be seen that air on inspiration from the anterior naris inlet begins to speed up in the nasal vestibule. Then most air flows through the middle and inferior turbinate after limen nasi and the air decelerates here. After posterior naris, the air arrives at the nasopharyngeal beginning in terms of linear flow. Since pharyngeal cross-sectional areas decrease, air accelerates quickly and passes to the outlet. For the laminar flow models in Fig. 4a,b, air flow is linear, and its streamline is regular in most parts of the upper airway. However, due to the sharp cross-sectional area decrease and shape alteration, some turbulence can be observed in the oropharyngeal part at a flow volume of 50 mL/s, enlarges greatly at 100 mL/s, and further aggravates until the simulation cannot converge at 200 mL/s using the laminar model. For the turbulent flow model, flow distributions and numerical values are approximately in accordance with the laminar model, but turbulence is clearer in the oropharyngeal part even at flow volumes of 50 and 100 mL/s, although their *Re* numbers in the whole flowing process are all smaller than 2100. Therefore, a turbulent CFD model is more suggested for flow characteristic research of the upper airway. If a laminar model is considered, its flow volume should be lower than 100 mL/s with an average *Re* number of 619. The laminar model is regarded as improper when the flow volume is up to 200 mL/s with an average *Re* number of 1248, which is much unlike the situation in classic fluid mechanics.

### Pressure distributions of different simulation models

**Figure 5:**
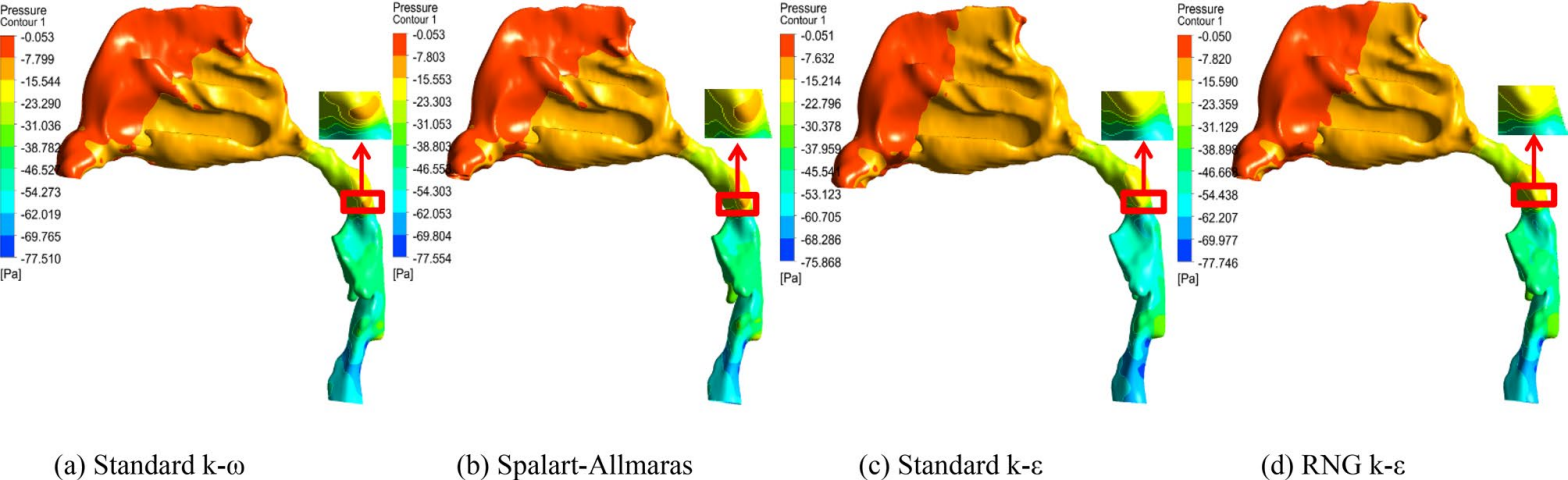
Pressure distributions of different turbulent flow models at a flow volume of 200 mL/s.

As shown in Fig. 5, the pressure distributions of different turbulent flow models at a flow volume of 200 mL/s are almost the same in terms of the pressure changing tendency and pressure drop value. The difference among the four simulations is that adverse pressure can be clearly observed in the oropharynx when CFD turbulent models are Standard k-ω and Spalart–Allmaras typles, whereas litter or even no adverse pressure occurs in Standard k-ε and RNG k-ε styles. Toward flow direction from nose to lung on inspiration time, normally its pressure will decrease gradually along upper airway. But due to a slight physiological expansion at the oropharyngeal beginning, flow velocity decreases and consequently pressure increases a little here. Therefore, the increased pressure is called as adverse pressure, which means that pressure increases in the oropharynx, but not decreases in accordance with normal flow and pressure distribution along upper airway. In classic fluid mechanics, the standard k-ω and Spalart–Allmaras models are better at simulating air flows of adverse pressure gradients, viscous near-wall regions and boundary layers, especially in low Reynold number flows. In contrast, the standard k-ε model is weak in handling complex flows with strong pressure gradients and is more suitable for high Reynolds number flows. Although RNG k-ε provides an analytical formula for flow viscosity of low Reynolds number, it is usually preferred in separate flow and secondary flow. Therefore, as the flow in the upper airway is of subsizing physiological structure, polytropic cross-section and low Reynolds number, standard k-ω and Spalart–Allmaras models are suggested when numerical analysis adopts the turbulent CFD method for flow characteristic research of the upper airway.

### Experimental validation and comparison with simulation

**Figure 6:**
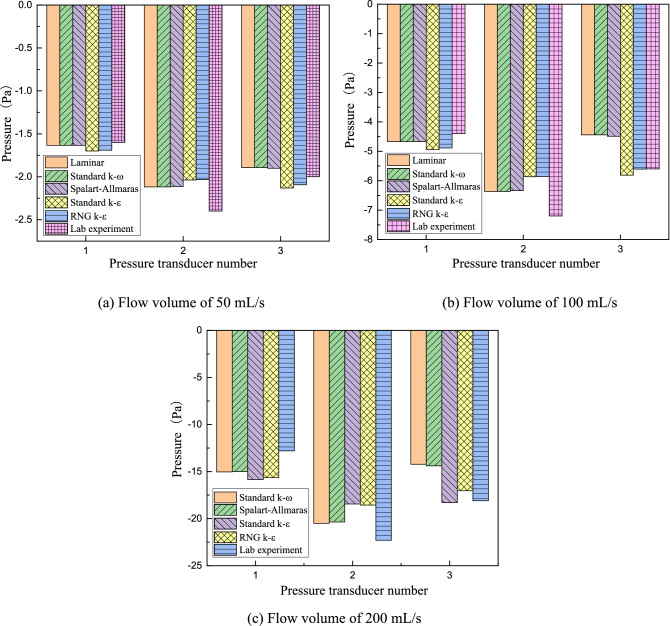
Comparisons of adverse pressure in CFD simulation and lab experiment.

The adverse pressures at three flow volumes are also detected in lab experiments by 3D printing technology. As shown in Fig. 6c, when the flow volume is 200 mL/s in the lab experiment, the oropharyngeal pressure measured by Transducer 3 is -18.1 Pa, larger than nasopharyngeal pressure of − 22.3 Pa (measured by Transducers 2). The adverse pressure difference is 4.2 Pa, which is approximately 18.9% of nasopharyngeal pressure. Correspondingly, in the simulation, the two pressures are -14.2 Pa and -20.5 Pa, respectively, and adverse pressure is 6.3 Pa, which is approximately 30.7% of nasopharyngeal pressure. The same phenomena occurrs at flow volumes of 50 and 100 mL/s shown in Fig. 6a,b. The error between the corresponding laboratory experiment and simulation result of different flow models at different flow volumes is approximately 20%, which can be caused by two aspects: (1) model reconstruction error from CT scanning images; (2) analysis neglecting the effect of some physiological structures, such as vibrissa resistance and neighboring soft tissue interactions. As the reliability and accuracy of laboratory experiments by 3D printing technology have been validated by real in vitro nasal resistance tests in our previous work, adverse pressure experiments can be regarded as reliable. Therefore, adverse pressure cannot be ignored because it can more precisely reflect real air flow in the upper airway.

## Further studies and discussions on adenoidal hypertrophy

According to the studies above, the next part of adenoidal hypertrophy analysis will adopt turbulent model of standard k-ω, and a boundary condition with inlet pressure of 0 Pa and outlet pressure of -20 Pa. Flow and pressure distributions of upper airway with and without OSAHS are also compared here.

### Model reconstruction with adenoidal hypertrophy

**Figure 7:**
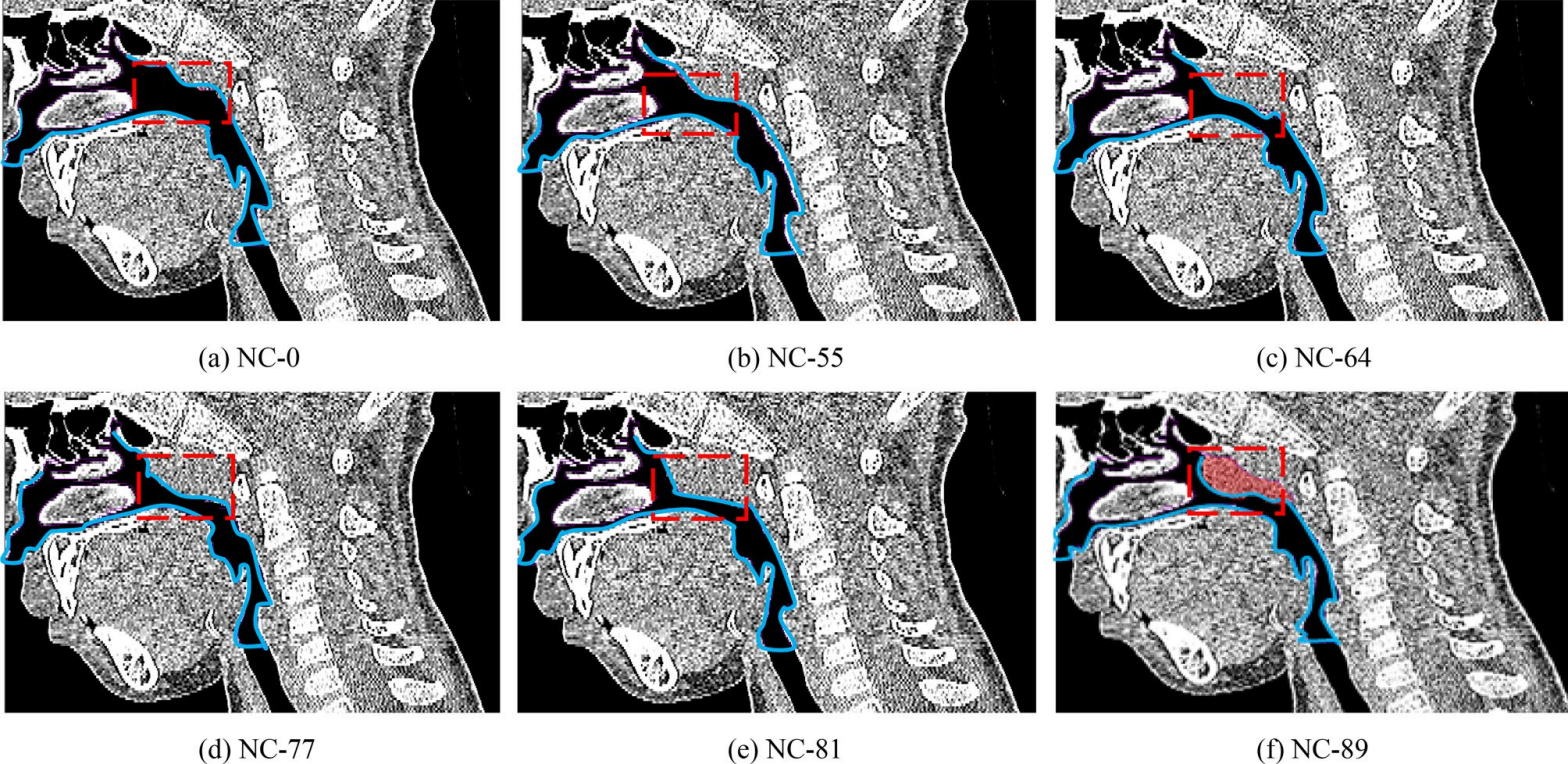
Illustration of different levels of adenoidal hypertrophy, where red wireframe represents adenoidal part, light blue wireframe represents upper airway, and light red shaded part in CT image (f) represents adenoidal hypertrophy.

Import CT image of the child with OSAHS described in Table 1 into Amira software, make a virtual operation by 1 mm issue resection on adenoid hypertrophy each time, and reconstruct eight upper airway models with different cross-section areas of adenoid (Cross-section 9) as shown in Fig. 7 and Table 3. Define a narrow coefficient as the index of different levels of adenoidal hypertrophy, which is shown as:

**Table 3:** Flow characteristics of different levels of adenoidal hypertrophy.

Narrow coefficient *NC*	Flow velocity *v*(m/s)	Cross-section area *A*_*i*_(× 10^–6^ m^2^)	Pressure drop *Δp* (Pa)	Volume flow rate *Q* (× mL/s)	Velocity coefficient *C*_*v*_	Flow discharge coefficient *C*_*d*_
0	0.83	104.8	3.60	77.81	0.351	0.314
31%	1.17	72.0	4.09	76.39	0.465	0.421
48%	1.56	54.2	4.89	76.13	0.567	0.510
55%	1.77	47.2	5.44	74.85	0.609	0.546
64%	2.14	37.7	6.37	68.54	0.681	0.578
77%	3.04	23.5	10.25	67.97	0.763	0.725
81%	3.40	19.6	11.78	63.08	0.796	0.753
89%	4.20	12.0	16.46	47.06	0.831	0.779

2 $$\mathrm{NC}=\left(1-\frac{{A}_{i}}{{A}_{0}}\right)$$

${A}_{i}$ is the area of cross-section 9 with different levels of adenoidal hypertrophy, and ${A}_{0}$ is the area of cross-section 9 without adenoidal hypertrophy. Therefore, *NC-31* represents that its adenoidal area of cross-section 9 is 31% of the area without adenoidal hypertrophy, and the number increases, the level of adenoidal hypertrophy is more serious. There is a clinical common on otorhinolaryngology finding that if *NC* arrives at approximately 66.7%, the child will be identified as pathological adenoid hypertrophy and needed an adenoidectomy criterion. For the child with OSAHS (Fig. 7f), his adenoidal area is approximately 12.0 mm^2^, and his *NC* is 89%, which means that this child is of a severe illness.

### Flow and pressure distributions with adenoidal hypertrophy

**Figure 8:**
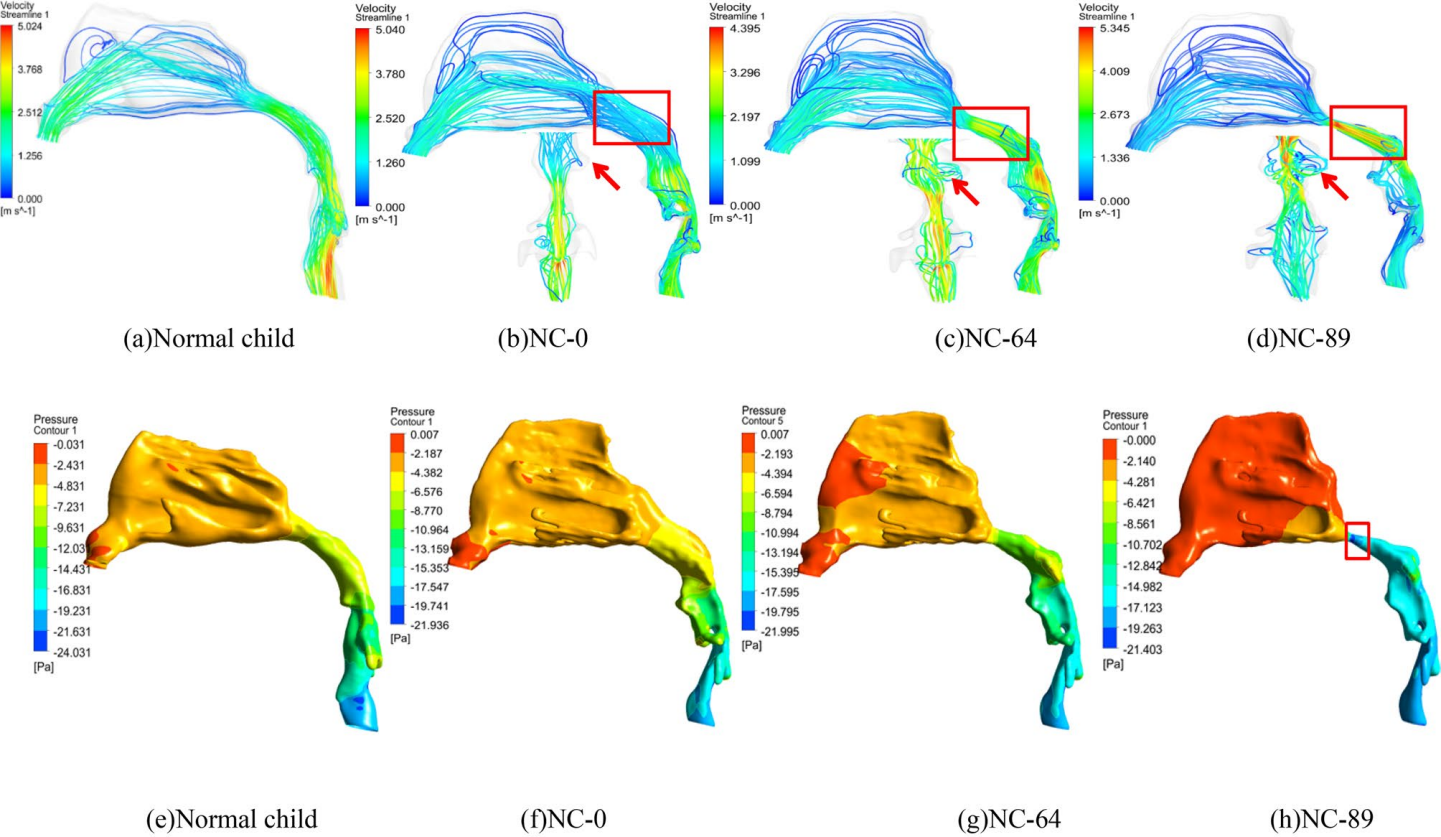
Flow and pressure comparisons of different adenoidal hypertrophies and normal children.

As shown in Fig. 8a-d and Table 3, when *NC* is 0, its flow distribution is almost similar with that of normal child. The air is gradually accelerated from the anterior naris inlet (cross-section 1) and its speed reaches the largest at the oropharyngeal part (between cross-sections 10 and 12). The whole stream line is almost regular and smooth. Until *NC* reaches 64%, the air speed at the nasopharyngeal part (between cross-sections 8 and 10) increases sharply with an increase of 0.082 m/s for an *NC* increase of 1%, which is approximately 4.8 times to that when *NC* is below 55%. In addition, the stream line also becomes disordered, and separate flows and large vortices begin to appear. Especially when *NC* exceeds 80%, the air speed at the adenoid (cross-section 9) becomes the largest in the upper airway, and a high-speed jet flow forms. The jet flow can produce a strong impact on the airway wall and cause a high-frequency flutter on neighboring soft issues in clinical syndromes, which will result in snoring, hypopnea and even sleep apnea.

The pressure distributions in Fig. 8 (e–h) and Table 3 again prove the syndrome. When *NC* is 0, its pressure distribution gradually decrease toward the flow direction, which is similar with that of normal child. Whereas *NC* is up to 64%, the air pressure at the adenoid (cross-section 9) decreases sharply with a decrease of 0.427 Pa for an *NC* increase of 1%, which is approximately 14 times to that when *NC* is below 55%. Especially when *NC* is 89%, its pressure drop between Cross-sections 8 and 9 is approximately 13.8 Pa, which is 69.2% of the whole pressure drop from inlet to outlet. The high pressure drop in the adenoidal part can strongly induce the shrinkage of neighboring soft issues or even the collapse, which will aggravate clinical syndrome.

### Flow characteristic analysis of upper airway

Because the cross-sectional area decreases and the pressure drop enlarges, the volume flow rate will decrease with increasing *NC*. As shown in Table 3, the flow volume initially decreases slowly and remains at a level of approximately 76 mL/s when *NC* is below 64%. Once *NC* reaches 64%, the flow volume decreases quickly from 74.85 mL/s at 55% to 68.54 mL/s and drops to 47.06 mL/s until the block reaches 89%. Medically, for a child with adenoidal hypertrophy, there is a qualitative judgment that if adenoid hypertrophy blocks 2/3 of the upper airway, it should be a medical intervention. The sharp decrease in flow volume when *NC* is above 64% can be a quantitative explanation for this operation index and the conclusion can be a useful and instructive principle for OSAHS mechanism and operation scheme enactment in clinical medicine.

Additionally, gained from the study are flow characteristic parameters: velocity coefficient and flow discharge coefficient. Note that the cross-sectional area of the adenoid (cross-section 9) decreases quickly in the upper airway; it can be assumed to be a throttling orifice, and its flow velocity $v$ and flow volume $Q$ can be calculated as:

3 $$\mathrm{v}={C}_{v}\sqrt{\frac{2\Delta p}{\rho }}\;(\text{m}/\mathrm{s})$$

4 $${\text{Q}} = {C_d}{A_i}\sqrt {\frac{{2\Delta p}}{\rho }} \;\left( {{{\text{m}}^3}/{\text{s}}} \right)$$

**Figure 9:**
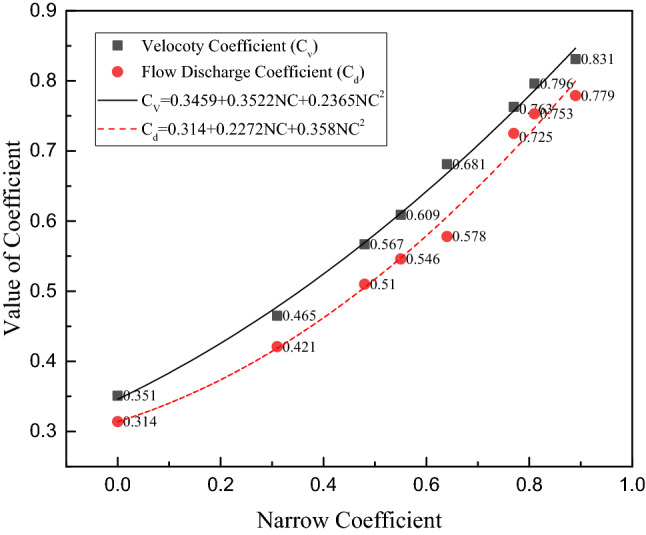
Velocity coefficient (*C*_*v*_) and flow discharge coefficient (*C*_*d*_) vs. narrow coefficient (*NC*).

${C}_{v}$ is the velocity coefficient, ${C}_{d}$ is the flow discharge coefficient, ${A}_{i}$ is the adenoid area of cross-section 9, $\Delta p$ is the pressure drop of cross-section 9 to cross-section 1 and $\rho$ is the air density of 1.29 kg/m^3^. From Fig. 9 and Table 3, it can be seen that with a narrow coefficient increase, the velocity coefficient and flow discharge coefficient both increase. Their relationships can be expressed by curve fitting equations:

5 $${C}_{v}=0.3459+0.3522NC+0.2365{NC}^{2}$$

6 $${C}_{d}=0.314+0.2272NC+0.358{NC}^{2}$$

In classic fluid mechanics, the flow discharge coefficient of the throttling orifice remains at a value of 0.5–0.6 if the flow is a full contraction flow, and if not, the coefficient will change with the Reynold number. Therefore, it can be concluded that upper airway flow is an incomplete contraction flow, and the structure of the upper airway plays a guiding role in air flow.

### Limitations and future work

The primary limitation of this paper is that there are a few volunteers in this research due to the considerations of privacy, psychological health or other scruples, and among the volunteers only two children are picked up meeting the requirements that whose age, sex, ethnicity, weight, and height are almost the same. This may question its broader validity for children of different ages.

Then the model is simplified under the conditions of inspiration time, awake status and no interaction with neighbor tissues, and does not include the effect of other factors, such as the influence of tonsillar hypertrophy, tidal breathing, interaction of neighbor tissues and so on, which certainly affect the flow characteristics of upper airway. Furthermore, more experiments can be carried out to prove the simulation, which can lead to more reliable results for clinical medicine. Therefore, a thorough study on different affecting factors and more compared subjects can be conducted in the future, and these are our next work and goals.

## Conclusions

This paper concentrates on the simulation method and flow characteristics of the upper airway for children with OSAHS. A 3D model of the upper airway starting from the anterior naris and ending at the trachea beginning is reconstructed based on CT images and solved in CFD to reveal its flow and pressure distributions. The *Re* number changes highly in the whole upper airway due to great cross-sectional area and shape alterations. As a result, turbulence can be clearly observed even at a low flow of 100 mL/s on inspiration with an average *Re* of 619, and the laminar assumption is no longer suitable for flow with a volume rate of 200 mL/s and an average *Re* number of 1248, as it cannot converge in this status, which is much unlike classic fluid mechanics. In addition, since the turbulent standard k-ε model and RNG k-ε model cannot figure out adverse pressure in the oropharynx, turbulent models of standard k-ω and Spalart–Allmaras are prior to suggestions for upper airway research. The pressure drop and adverse pressure of different flow models and volume rates in the simulation are validated in lab experiments by 3D printing technology with an error of approximately 20%. Additionally, carried out in this analysis is the influence of adenoidal hypertrophy with different *NC* levels. When *NC* is above 64%, the inner flow field will change greatly at the nasopharyngeal part with the appearance of strong turbulence, formation of high-speed jet flow and high pressure drop, and consequently strong shrinkage on the airway wall and high-frequency flutter on neighboring soft issues will appear, which can result in snoring, hypopnea and even sleep apnea in clinical syndrome. The flow volume will decrease quickly from 74.85 mL/s at 55% NC to 68.54 mL/s at 64% NC and drop to 47.06 mL/s at 89% NC, which can be a quantitative explanation for medical intervention if adenoid hypertrophy blocks 2/3 of the upper airway in the clinical common judgment of otorhinolaryngology. It is expected that this paper can provide a further understanding of the OSAHS pathology mechanism and meaningful instruction on surgery plan making as well as recovery evaluation postoperatively.
